# A274 THE EFFECT OF DIETARY MODULATION ON FUNGAL AND VIRAL COMMUNITIES IN THE GASTROINTESTINAL TRACT: A LITERATURE REVIEW

**DOI:** 10.1093/jcag/gwac036.274

**Published:** 2023-03-07

**Authors:** J Buttar, E Kon, S Lawal, H Armstrong, B Bressler, K Jacobson, G Healey

**Affiliations:** 1 Internal Medicine; 2 University of British Columbia, Vancouver; 3 Medical Microbiology and Infectious Diseases; 4 Assistant Professor -- Medical Microbiology and Infectious Disease , University of Manitoba, Winnipeg; 5 Clinical Associate Professor of Medicine -- Gastroenterology, University of British Columbia; 6 Pediatric Gastroenterology, BC Children's Hospital Research Institute; 7 Department of Pediatrics, University of British Columbia, Vancouver, Canada

## Abstract

**Background:**

The human gut microbiome is a complex, dynamic community that has been shown to impact gastrointestinal (GI) diseases, including inflammatory bowel disease (IBD). Particularly, changes in diet quality and composition can influence the composition and function of the gut microbiome, leading to an inappropriate response to inflammation, increasing risk for IBD flare. Currently, our understanding of diet modulation largely revolves around changes in bacterial composition and diversity, despite our knowledge of neighbouring eukaryotes, viruses, and archaea within the GI tract. This literature review serves to bolster our understanding of diet modulation on viral and fungal communities, in an effort to support non-pharmacological therapies in IBD.

**Purpose:**

To assess current literature investigating the effect of diet modulation on the gut microbiota, with interest in viral and fungal communities.

**Method:**

We performed a MEDLINE and EMBASE literature review with the following search terms: "microbiome / microbiota / microbe" and "fungi / fungal" or "virus / viral / virome" and "diet composition" or "high fiber/re diet" or "diet therapy" or "diet."

Exclusion criteria:

- Abstracts

- Articles not in English

- Expert Review articles

- Articles investigating bacterial microbiota communities exclusively

**Result(s):**

Our EMBASE and MEDLINE search identified 418 and 343 articles, respectively. After applying our exclusion criteria and removing duplicates, 13 articles were included (5 - fungal communities exclusively, 4 - viral communities exclusively, 4 - both).

Novel research directly investigating the impact of diet modulation on fungal and viral communities in the gut microbiota are exceedingly rare. Despite this, both communities in animal and human models showed increases in diversity in response to diet modulation, independent of changes in bacterial composition.

Fungal communities in animal studies showed a four-fold increase in diversity with changes in diet composition (standard chow to high-grain/ high fibre /low fat) as soon as 28 days post dietary commencement. In human studies, fungal communities changed in response to short term diet changes, not long-term. Candida was positively correlated with carbohydrate ingestion, while Aspergillus was negatively correlated with short chain fatty acid ingestion.

Viral communities in animal models showed a similar increase in diversity in response to diet modulation (standard chow to high-grain/high-fibre). In human studies, virome response to diet increased most significantly in those with lower initial viral diversity. However, viral diversity was impacted most by interpersonal variation, not diet modulation.

**Conclusion(s):**

Diet modulation remains a key player in altering the gut microbiome, extending to fungal and viral communities. Further studies are required to elucidate the magnitude and temporal effect on these lesser studied microbial communities, given recent studies showing the importance of fungal/viral microbes in IBD.

**Please acknowledge all funding agencies by checking the applicable boxes below:**

None

**Disclosure of Interest:**

None Declared

NEUROGASTROENTEROLOGY & MOTILITY

